# Surface Engineering of Top7 to Facilitate Structure Determination

**DOI:** 10.3390/ijms23020701

**Published:** 2022-01-09

**Authors:** Yuki Ito, Takuya Araki, Shota Shiga, Hiroyuki Konno, Koki Makabe

**Affiliations:** Graduate School of Science and Engineering, Yamagata University, 4-3-16 Jyonan, Yonezawa 992-8510, Yamagata, Japan; tdc43096@st.yamagata-u.ac.jp (Y.I.); twy26391@st.yamagata-u.ac.jp (T.A.); tye31578@st.yamagata-u.ac.jp (S.S.); konno@yz.yamagata-u.ac.jp (H.K.)

**Keywords:** Top7, surface entropy reduction, crystal packing engineering

## Abstract

Top7 is a de novo designed protein whose amino acid sequence has no evolutional trace. Such a property makes Top7 a suitable scaffold for studying the pure nature of protein and protein engineering applications. To use Top7 as an engineering scaffold, we initially attempted structure determination and found that crystals of our construct, which lacked the terminal hexahistidine tag, showed weak diffraction in X-ray structure determination. Thus, we decided to introduce surface residue mutations to facilitate crystal structure determination. The resulting surface mutants, Top7sm1 and Top7sm2, crystallized easily and diffracted to the resolution around 1.7 Å. Despite the improved data, we could not finalize the structures due to high R values. Although we could not identify the origin of the high R values of the surface mutants, we found that all the structures shared common packing architecture with consecutive intermolecular β-sheet formation aligned in one direction. Thus, we mutated the intermolecular interface to disrupt the intermolecular β-sheet formation, expecting to form a new crystal packing. The resulting mutant, Top7sm2-I68R, formed new crystal packing interactions as intended and diffracted to the resolution of 1.4 Å. The surface mutations contributed to crystal packing and high resolution. We finalized the structure model with the R/R_free_ values of 0.20/0.24. Top7sm2-I68R can be a useful model protein due to its convenient structure determination.

## 1. Introduction

Top7 is a de novo designed protein consisting of two α-helices and five β-strands [1]. Because of the lack of an evolutional trace, Top7 is an ideal model protein to study the pure nature of protein structure. The high stability of Top7 also provides a suitable property for protein engineering. For example, the replacement of hydrophobic core residues to polar hydrogen-bonded residues in this protein has been used to evaluate the effect of an “inside out” protein design, which could be stable in organic solvents [2]. The stable nature of the Top7 scaffold was also used for grafting a target binding loop [3,4] or an antigenic epitope [5,6].

**Figure 1:**
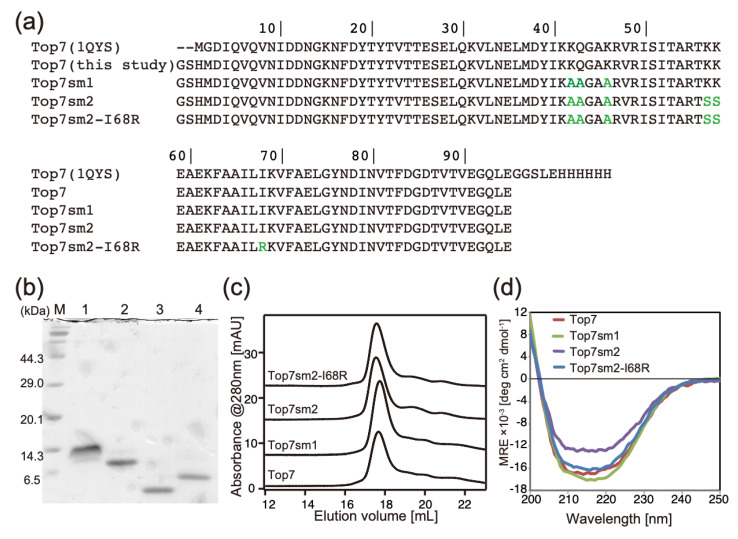
(**a**) Amino acid sequences of Top7 and its variants used in this study. Mutated residues are shown in green characters. (**b**) Purified protein samples analyzed by SDS-PAGE. M: molecular-weight marker, 1: Top7, 2: Top7sm1, 3: Top7sm2, 4: Top7sm2-I68R. (**c**) Analytical size-exclusion chromatography. Chromatograms at 280 nm absorbance were shown. Each chromatogram is shifted vertically for clarity. (**d**) Far-UV CD spectra of TOP7 surface mutants.

We had started a protein engineering project using Top7, and we tested the structure determination process of Top7 by X-ray crystallography, which reported previously [1]. Unfortunately, our attempt to determine the crystal structure failed. We tested not only the reported crystallization condition but also 192 crystal screening conditions. We found that the protein forms nice single crystals in several screening conditions, but they diffracted poorly at a synchrotron facility. Therefore, we could not obtain diffraction data beyond a resolution of ~3.0 Å. Considering the fact that the original Top7 structure was determined to the resolution of 2.5 Å, sequence differences between the original protein and our construct may explain this discrepancy (Figure 1a). Our construct had an N-terminal hexahistidine tag that was later removed by protease during a purification step, while the original construct contained a C-terminal hexahistidine tag. It is also possible that the Top7 crystals show varied diffraction quality, and testing many numbers of crystals may be required to obtain the ~2.5-Å resolution data because we found our Top7 crystals showed varied resolution from ~8 Å to ~3.5 Å. Thus, it is important to develop a Top7 variant whose structure can be determined easily to facilitate the Top7 engineering research. Here, we aimed to develop a Top7 mutant whose structure can be easily determined with better resolution via a surface entropy reduction approach, which replaces large flexible residues on the protein surface to smaller ones to promote crystallization packing [7,8,9,10]. Eventually, we successfully determined a surface mutant structure of Top7 at a resolution of 1.4 Å. We will further discuss the structural details below.

## 2. Results and Discussion

### 2.1. Mutant Constructions and Preparation

To determine the molecular structure of Top7, we chose the high-entropy surface residues Lys42, Gln43, and Lys46 to apply the surface entropy reduction method. We introduced K42A, Q43A, and K46A mutations and the resulting mutant was named Top7sm1 (Figure 1a; sm indicates surface mutations). Furthermore, we chose additional high entropy residues, Lys57 and Lys58. We mutated these two residues to serine and called this mutant Top7sm2. Three residues of Top7sm1 were mutated to low entropy alanine to reduce the conformational entropy loss during crystal contact formation. For the additional two mutations of Top7sm2, serine mutations were introduced instead of alanine to prevent the surface from being too hydrophobic.

Top7sm1 and Top7sm2 were expressed in *E. coli* and purified by a one-step Ni-NTA purification. After removal of the N-terminal hexahistidine tag, purified protein samples were obtained (Figure 1b). Although the chain lengths of these mutants are identical, the mutations altered the number of positively charged residues. This could yield different mobilities during electrophoresis.

### 2.2. Size-Exclusion Chromatography and CD Measurements

Size-exclusion chromatography (SEC) was performed to measure the size of the protein samples (Figure 1c). All surface mutants were eluted as a monomer at a similar elution volume to Top7, indicating that the surface mutations did not cause the intermolecular association. CD measurements evaluated the secondary structure and demonstrated that the surface mutants showed similar CD profiles at far-UV regions with that of wildtype. This indicates that the secondary structure was not affected by the surface mutations (Figure 1d). Taken together, these results confirm a minimal structural impact, resulting from the mutated surface residues.

### 2.3. Structure Determination of Top7sm1 and Top7sm2

**Figure 2:**
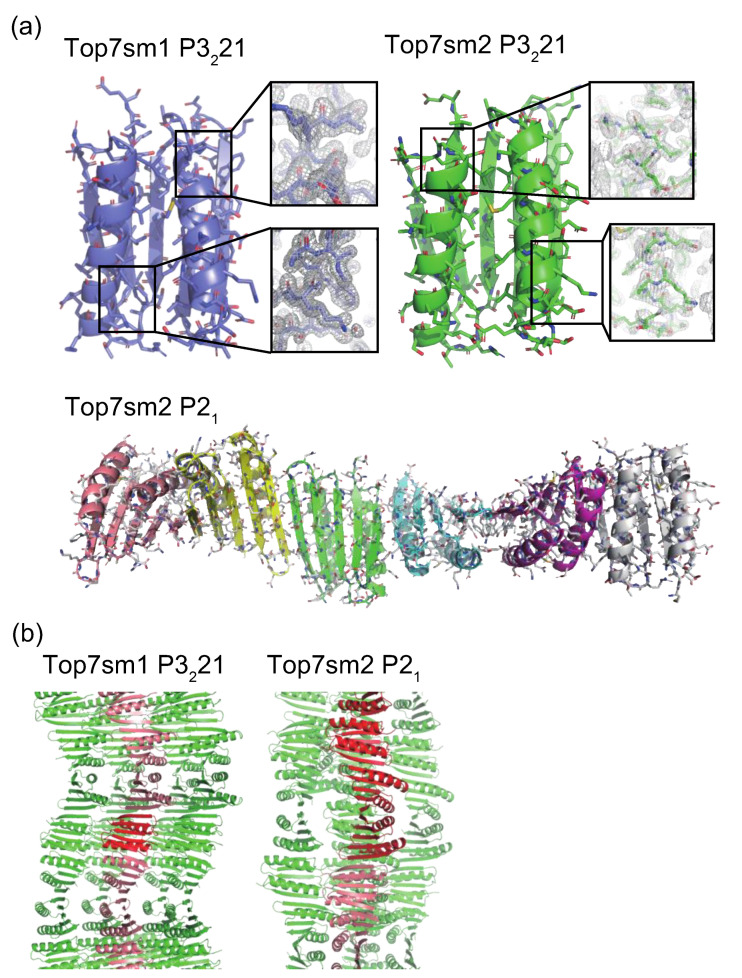
Crystal structures of Top7sm1 and Top7sm2. (**a**) Protein structures in asymmetric units are shown with cartoon and stick representations. Six monomers in the asymmetric unit of the Top7sm2 P2_1_ crystal are shown by six different colors. Electron density maps (contour level of σ = 1.0) for P3221 crystals are shown in rectangles with mesh representations. (**b**) Crystal packings of Top7sm1 P3_2_21 and Top7sm2 P2_1_. Proteins in the asymmetric units are shown in red. One-directional arrays via intermolecular β-sheet are indicated with salmon color of symmetry-related molecules.

We successfully crystallized Top7sm1 and Top7sm2 and obtained diffraction data at a resolution of 1.7 Å, 1.65 Å, and 2.5 Å for TOP7sm1 with the P3_2_21 space group, Top7sm2 with the P3_2_21 space group, and Top7sm2 with the space group P2_1_, respectively. Two P3_2_21 data points were isomorphous with that of the original Top7 structure (PDBID 1QYS), and one P2_1_ datum of Top7sm2 contained six molecules in an asymmetric unit (Figure 2a). Since two P3_2_21 crystals had moderate resolutions, we could see relatively clear electron density maps for these mutants (Figure 2a). However, despite the high quality of the diffraction data of the P3_2_21 crystals, the R values remained high (R/R_free_ = 0.26/0.29 and 0.26/0.29 for Top7sm1 and Top7sm2, respectively). Considering their resolution, these values were beyond the acceptable range of structure refinement. Although we have tested several refinement strategies, we could not improve the R values. The R values for the Top7sm2 P2_1_ mutant were also a bit high, even considering its resolution (2.5 Å; R/R_free_ = 0.27/0.31). Due to the high R values, we could not finalize the structure models to fulfill the deposit criteria for the Protein Data Bank (PDB). We analyzed these incomplete models to evaluate the reason for the high R values. PDB files of these mutants (Top7sm1 and Top7sm2) can be found in the supplemental materials.

All Top7 variants shared a very similar structure with Top7 with the root mean square deviation (RMSD) values of Cα atoms below 0.8 Å. The Top7sm1 and Top7sm2 structures with the P3_2_21 space group showed isomorphous crystals with the original Top7 structure. It appears that the surface mutations did not alter the crystal packing. It is possible that removal of positive charges of lysine residues may contribute to the improved resolutions, electrostatically. Top7sm2 structure with the P2_1_ space group contained six molecules within the asymmetric unit. Interestingly, these six molecules formed a consecutive string via an intermolecular β-sheet (Figure 2a). Such consecutive strings of the β-sheet also exist in the P3_2_21 crystals generated by symmetry operations (Figure 2b). Thus, all the variant structures share the common one-directional β-sheet string via intermolecular association. Although the reason for the high R values of data of the Top7 variants is not clear thus far, it may be possible that with crystal packing alterations, we could obtain new crystal forms with improved refinement statistics.

### 2.4. I68R Mutation on Top7sm2 to Disrupt the Continuous β-Sheet in Crystal Packing

**Figure 3:**
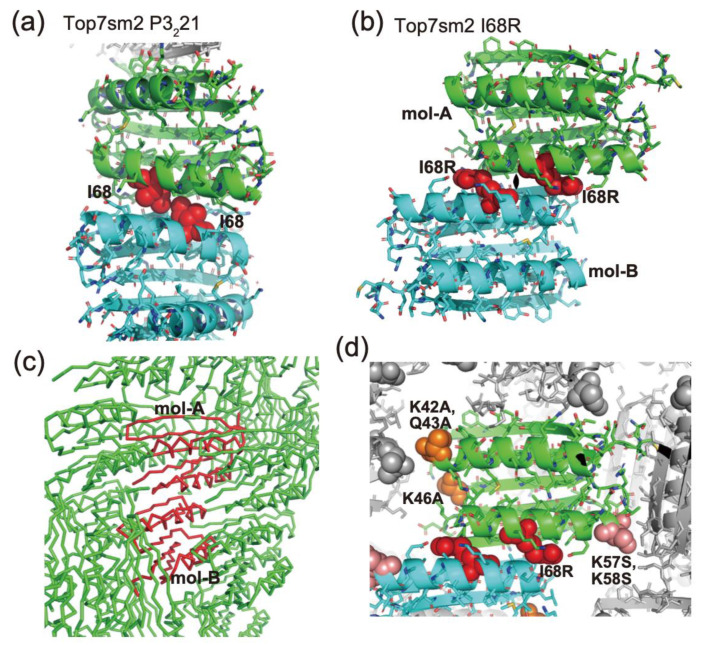
Mutant design and crystal structure of Top7sm2-I68R. (**a**) Intermolecular packing of Top7sm2 P3_2_21, which is commonly shared in other Top7 crystals. I68 residues are shown in red with sphere representations. (**b**) Crystal structure of Top7sm2-I68R. Two molecules in the asymmetric unit are shown (mol-A: green, mol-B: cyan). Mutated R68 residues are shown in red with CPK representations. (**c**) Crystal packing of Top7sm2-I68R structure. Two molecules in the asymmetric unit are shown in red ribbon representation. (**d**) Close view of the crystal packing. Surface mutations in sm1 (K42A, Q43A, and K46A) are shown in orange and those in sm2 (K57S and K58S) are shown in pink. Symmetry-related molecules are shown in gray.

To obtain the different crystal packing of Top7 variants, we mutated the intermolecular residue to disrupt the consecutive β-sheet observed in all Top7 structures. We scrutinized the crystal packing of Top7sm2 and found Ile68 at β-strand-4 to be a promising site for the mutation. Ile68 is located where an intermolecular β-strand and a symmetry-related molecule interact (Figure 3a). We mutated Ile68 to arginine to introduce repulsive electrostatic force by positive charges and steric hindrance at the interface. The resulting mutant, Top7sm2-I68R, was monomeric in solution as confirmed by SEC. This mutant also showed a similar CD profile to that of Top7 (Figure 1c,d).

We successfully determined the crystal structure of Top7sm2-I68R with a resolution of 1.4 Å (Figure 3b and Table 1). The crystal belongs to the P2_1_ space group, containing two molecules in the asymmetric unit (mol-A and mol-B). The two molecules had very similar structure with an RMSD value of Cα atom as 0.36 Å. As intended, the mutant formed new crystal packing without forming the continuous β-sheet string that was observed in the other Top7 structures (Figure 3c), and the R values (R/R_free_: 0.20/0.24) were dropped to a relatively decent range for the resolution. The structure was deposited to PDB with a PDBID of 7FAO.

Two molecules in the asymmetric unit were positioned in a side-by-side manner with a pseudo-twofold symmetry (Figure 3b). Arg68 slid by each other to avoid steric crash and repulsive electric force, as intended. The surface mutation residues were located near the crystal contact regions (Figure 3d). Although there is no direct contact at K42A, Q43A, and K46A, these mutations seem to prevent steric crash with a neighboring molecule by the removal of the long Lys and Gln side chains. K57S and K58S mutational portions directly form a crystal contact with the Glu28 of a neighboring molecule. Such favorable influences of the surface mutations should contribute to the improvement of the resolution and reduction of the R values.

It has been reported that the resolution improvement can be achieved by the surface engineering of protein. For example, RhoGDI and IGFRK-0P structures received improved resolutions from 2.0 Å to 1.2 Å and 2.7 Å to 1.5 Å, respectively, via surface mutations [11,12]. The critical difference of our approach is aiming for alternating crystal packing by introducing a bulky charged residue onto the symmetric crystal contact position to alternate the crystal packing because the original crystal packing of Top7 induces continuous intermolecular β-sheet and may result in unfavorable statistical values. Thus, an intentional insertion of a disruptive mutation on crystal packing as shown in this study could be a useful strategy to obtain different type of crystal packings.

### 2.5. Top7sm2-I68R as a Model Protein

We successfully constructed the Top7 surface mutant (Top7sm2-I68R) that easily forms a single crystal and has a different crystal packing structure from the original Top7. We could expand the resolution from 2.5 Å of the original Top7 to 1.4 Å by surface engineering. The original Top7 structure model (PDBID 1QYS) lacks several side chain atoms, while the Top7sm2-I68R structure contains all side chain atoms because of the high-resolution data. Easy structure determination and the resolution improvement are important for structural analysis investigating the molecular detail of further engineered Top7.

We noticed that the molecular surface of Top7 has a relatively high number of charged bulky residues (Lys and Glu), possibly resulting from the de novo designing procedure. Thus, surface engineering for de novo designed proteins may be a feasible approach for general crystal structure determination.

## 3. Materials and Methods

### 3.1. Sample Preparations

A synthetic gene of Top7 was purchased from Eurofins genomics (eurofinsgenomics.jp; accessed on 20 October 2021) and subcloned into a pET28 vector. Expression vectors of the Top7 variants were constructed by a PCR-based mutagenesis using a PrimeSTAR DNA polymerase (Takara Bio Inc., Shiga, Japan). All protein samples were expressed by *E. coli* BL21(DE3) strain using Studier’s autoinduction medium [13]. After one-step purification with Ni-NTA agarose resin (FUJIFILM Wako Pure Chemical Corporation, Tokyo, Japan), the N-terminal hexahistidine tag was removed by thrombin cleavage. The protein samples were re-applied to the Ni-NTA column to remove the cleaved tag and uncleaved protein.

Analytical SEC was performed using a Superdex200 10/300 column (GE Healthcare, Chicago, IL, USA) equilibrated with a 50 mM phosphate pH 7.0, 150 mM NaCl buffer by monitoring 280 nm absorbance.

CD spectra were measured using a CD spectrometer J-820 (JASCO, Tokyo, Japan) with 10 μM protein samples in a 50 mM phosphate pH 7.0, 150 mM NaCl buffer, and 25 °C. A 1 mm quartz cell was used.

### 3.2. Crystallization and Structure Determination

Initial crystallization conditions were screened using Crystal screen 1/2 and JCSG+ (192 conditions in total). Hanging drop vapor diffusion was used for crystallization. Protein crystals were formed in conditions of 20% PEG3350, 0.25 M ammonium formate (pH 6.6) for Top7sm1 (26 mg/mL protein concentration), 12% PEG8000, 12% glycerol, 0.16 M calcium acetate, 0.1 M cacodylic acid (pH 6.5) for Top7sm2 (25 mg/mL protein concentration), and 15% PEG8000, 0.1 M magnesium acetate, and 0.1 M cacodylic acid (pH 6.5) for Top7sm2 I68R (25 mg/mL protein concentration). As a cryoprotectant, glycerol was added to the crystallization buffer at concentrations of 15~30%. We collected diffraction data at Photon Factory beamline BL-5A and processed with xds [14]. For the initial phase determination, the Top7 structure was used (PDBID 1QYS). Structural refinements were performed using Phenix [15] and Coot [16]. The final structural model of Top7sm2-I68R was deposited to PDB with the PDBID of 7FAO. PDB format files for Top7sm1 and Top7sm2 are available as supplemental files. Molecular visualization was shown by PyMol (www.pymol.org; accessed on 20 October 2021).

## Figures and Tables

**Table 1:** Statistics for the crystal structure.

Protein	Top7SuMu2 I68R
Data collection statistics	
Space group	P 1 2_1_ 1
Cell parameters	a = 24.02
	b = 86.30
	c = 40.13
	β = 97.18
Beamline	KEK-PF BL5A
Wavelength	1.0000
Resolution (Å) ^a^	19.91–1.43
Completeness(%)	98.64 (99.46)
I/s (I)	17.0 (2.5)
R_merge_ ^b^	0.034
Average redundancy	3.2(3.2)
Refinement statistics	
Resolution range (Å)	19.908–1.430 (1.481–1.430)
Reflections used (free)	29,515 (2957)
R factor ^c^	0.2029
R_free_^d^	0.2422
RMS deviations	
Bonds (Å)	0.005
Angles (°)	0.73
No. protein residues	96
No. waters	127
Ramachandran plot statistics	
Favored (%)	98.94
Allowed (%)	1.06
Outliers (%)	0.00

^a^ Highest resolution shell is shown in parenthesis. ^b^ R-merge = Σ_hkl_Σ_i_| I(hkl)_i_ − <I(hkl)>| /Σ_hkl_Σ_i_<I(hkl)_i_> over *i* observations of a reflection hkl. ^c^ R-factor = Σ||F(obs)| − |F(calc)||/Σ|F(obs)|. ^d^ R_free_ is R with 5% of reflections sequestered before refinement.

## Data Availability

Structure data for Top7sm2-I68R is available at PDB with the ID of 7FAO.

## Supplementary Materials

The following are available online at https://www.mdpi.com/article/10.3390/ijms23020701/s1.
